# Accelerated degradation of plastic products via yeast enzyme treatment

**DOI:** 10.1038/s41598-023-29414-1

**Published:** 2023-02-10

**Authors:** Hiroko Kitamoto, Motoo Koitabashi, Yuka Sameshima-Yamashita, Hirokazu Ueda, Akihiko Takeuchi, Takashi Watanabe, Shun Sato, Azusa Saika, Tokuma Fukuoka

**Affiliations:** 1 National Agriculture and Food Research Organization (NARO), Tsukuba, Japan; 2 Research Institute for Sustainable Chemistry, National Institute of Advanced Industrial Science and Technology (AIST), Tsukuba, Japan; 3 Present Address: Gunma Industrial Technology Center, 884-1 Kamesato, Maebashi, Gunma 379-2147 Japan

**Keywords:** Biotechnology, Environmental sciences, Materials science

## Abstract

Biodegradable plastics can solve the problem of unwanted plastics accumulating in the environment if they can be given the contradictory properties of durability in use and rapid degradation after use. Commercially available agricultural biodegradable mulch films are made from formulations containing polybutylene adipate-*co*-terephthalate (PBAT) to provide mechanical and UV resistance during the growing season. Although used films are ploughed into the soil using a tiller to promote decomposition, it is difficult if they remain durable. We showed that an enzyme produced by the leaf surface yeast *Pseudozyma antarctica* (PaE) degrades PBAT-containing films. In laboratory studies, PaE randomly cleaved the PBAT polymer chain and induced erosion of the film surface. In the field, commercial biodegradable films containing PBAT placed on ridges were weakened in both the warm and cold seasons by spraying the culture filtrate of *P. antarctica*. After the field was ploughed the next day, the size and total weight of residual film fragments decreased significantly (*p* < 0.05). Durable biodegradable plastics used in the field are degraded using PaE treatment and are broken down into small fragments by the plough. The resultant degradation products can then be more readily assimilated by many soil microorganisms.

## Introduction

Plastic products for outdoor use, including agricultural use, can improve productivity, but the problems associated with their accumulation and dispersal in the environment must be resolved. Biodegradable plastics (BPs) are a promising solution. However, the durable BP products that can replace existing non-degradable products still degrade very slowly in open environments. If BP polymer chain cleavage can be intentionally promoted after the BP is used, the resulting oligomers can be assimilated by various microorganisms in the environment, promoting a BP-derived carbon cycle and preventing accumulation^1^.

Mulch films are used in agriculture to cover field surfaces as they stabilise moisture and soil temperature, prevent soil erosion and weed growth, promote plant growth, and enable high quality crop production while reducing the demand for water, herbicides, and fertilisers, thereby contributing to sustainable agriculture^2^. The worldwide consumption of traditional non-degradable polyethylene mulch film is 1.8 million tonnes^3^. However, it is difficult to recover after use, and unrecovered residual film pieces accumulate in the environment^2–9^. Even when time and effort are expended to collect mulch films, they are difficult to recycle because of high soil contamination. In 2019, the recycling rate of non-degradable mulch in EU was 0, and currently, the waste is either incinerated for heat recovery or is buried in landfills^10^.

Several decades ago, biodegradable mulch films made of aliphatic polyesters—poly(butylene succinate-*co*-adipate) (PBSA) and poly(butylene succinate) (PBS)—were developed, that completely decompose after ploughing into soil. Recently introduced mulch films contain poly(butylene adipate-*co*-terephthalate) (PBAT), an aromatic polyester, which improved their durability while maintaining the mulching function^9,11,12^. The current use of biodegradable mulch remains low; however, as its usage increases^7^, the use of films containing PBAT will increase accordingly. Although PBAT can be composted^13^, it degrades slowly in the soil^14^, and the degradation rate of PBAT compounding film is considerably slower in the field^11,12^. Therefore, a new method is required to accelerate the degradation of used films and reduce the release of film fragments into the environment^7^.

During the degradation of BP in an open environment, the release of low-molecular-weight organic compounds from polymers by chemical, physical, and enzymatic activities is a slower rate-limiting step than the next step, in which microorganisms take up small molecules and break them down into water and carbon dioxide^7^. Therefore, treatment with a polyester-degrading enzyme^15^ was considered to accelerate the entire process. Previously, it was reported that PBAT degradation enzymes decrease the turbidity of the PBAT powder or films under optimal conditions *in vitro*^16–20^. A cutinase-like enzyme from the filamentous fungus *Paraphoma* sp. B 47 -9 (PCLE) has the ability to break down PBAT, PBSA, and PBS^18^. We applied commercial mulch films containing PBAT, PBSA, and PBS (monomer-based weight ratio of 17:39:44, commercial film A) to the ridges of the field and then treated the surface with a combination of moisturizer powder, carboxymethylcellulose, and PCLE. This treatment caused the films to crack on the following day^21^. Since then, commercial films that are tougher have been developed, with improvements such as a higher PBAT content and sometimes the addition of durable compostable polylactic acid (PLA). However, because PLA is not degraded by PCLE^18^, an enzyme that can degrade various types of films is needed. An enzyme produced by the leaf-surface yeast *Pseudozyma antarctica* (PaE) can degrade amorphous PLA film^22–24^. It is also more active than PCLE in cleaving the polymer chains of PBS and PBSA films^25^. Furthermore, in laboratory incubation experiments, PaE-treated films degraded faster when embedded in soil. Pieces of commercial film A (2 × 2 cm) were coated with a PaE solution and allowed to dry on a bench. The next day, they were buried in a petri dish containing soil. The films were removed every week for tests, and the enzyme-treated films lost their shapes faster than the untreated ones^26^. However, the ability of PaE to degrade PBAT has not yet been demonstrated. Enzymes that are highly active in breaking down plastic materials may accelerate the degradation of films that blend these materials.

In this study, the ability of PaE to degrade PBAT was evaluated in the laboratory. Commercial mulch films with different blends of PBAT were placed on the ridges of the field and then treated with PaE to investigate its effects on the degradation of the films. Therefore, a number of methods were developed to assess the degradation of BP mulch films in the field.

## Results

### Degradation of PBAT and blended films

**Figure 1:**
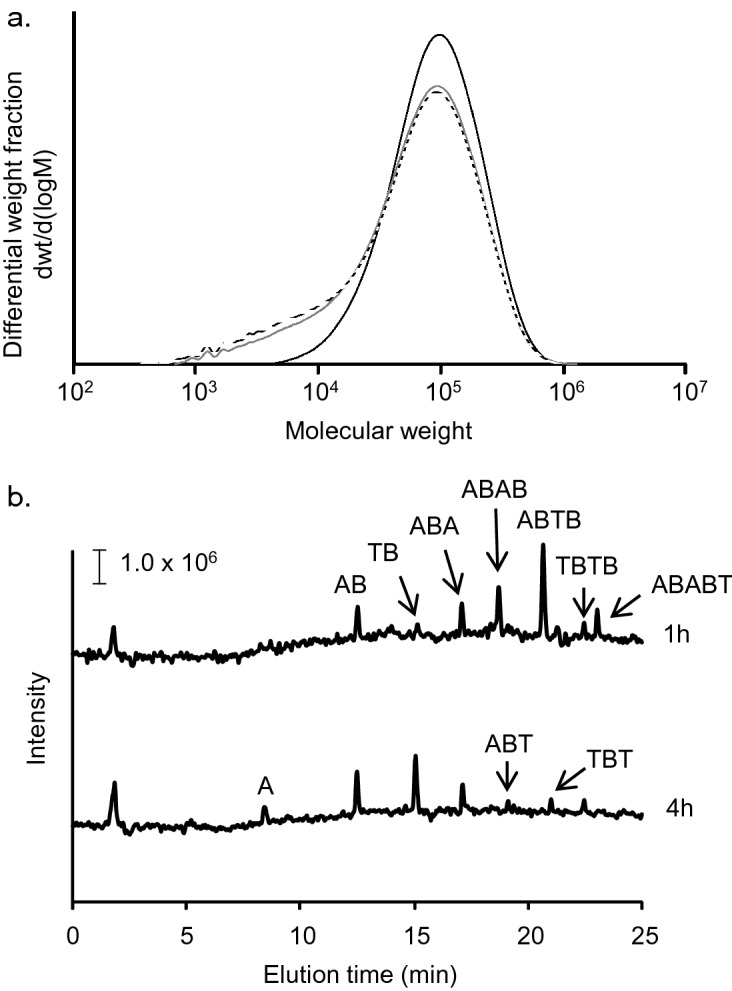
Degradation profile of PBAT cast film treated with PaE. (**a**) Molecular weight distribution of PBAT in films analysed using size-exclusion chromatography (SEC) after PaE-catalysed degradation of PBAT cast film for 4 h (grey line) and 24 h (dashed line). The black line shows the molecular weight distribution of residual films after incubation without PaE (buffer only) for 24 h. (**b**) Total ion current chromatogram ( −) of the water-soluble products of PaE-catalysed degradation of PBAT cast film for 1 and 4 h examined by LC–MS. A, adipate; B, butanediol; T, terephthalate.

In the present study, we first determined whether purified PaE degrades cast PBAT films on a slide glass. The proportion of residual polymers with a molecular weight distribution of less than 10^4^ Da increased four and twenty-four hours after the reaction (Fig. 1a). This resulted in changes in the average molecular weight (Mn) and polydispersity (Mw/Mn) from Mn = 2.33 × 10^4^ Da and Mw/Mn = 4.5 (4 h) to Mn = 1.85 × 10^4^ and Mw/Mn = 5.4 (24 h). Figure 1a also shows that there was no shift at the peak of the molecular weight distribution after the reaction proceeded, meaning that there was little change in the average molecular weight (Mw) of 10.4 × 10^4^ Da (4 h) and 10.0 × 10^4^ Da (24 h). Various oligomers formed by polymer chain scission were eluted in the enzyme reaction solution (Fig. 1b). With increasing reaction time (1–4 h), small oligomers increased, and the PBAT monomers adipic acid (Fig. 1b) and butanediol (Supplementary Fig. S1) were also detected. Subsequent experiments were performed using either *P. antarctica* culture filtrates or dilutions prepared from them. When heat-pressed sheets (pieces 3 × 3 cm) were immersed in 1.1 µM PaE in a buffer (pH 8.0) at 30 °C for 3 h, the sheet was degraded in the order PBSA > PBS > PBAT, and the weight loss per h of each sheet was 45.2 mg, 9.3 mg, and 2.3 mg, respectively (Supplementary Table S1). The pH of the reaction solution that the PBAT film was immersed in for 3 h decreased slightly to 7.86 (STEV = 0.02). The interior of the PBAT polymer chain was randomly cleaved via an endo-type attack by PaE and broken down into monomers, which were then scraped from the film surface. Similarly, weight loss in commercial BP mulch films A and B, which contained PBAT, PBSA, and PBS at a monomer-based weight ratio of 17:39:44 and 90:0:10, was 4.7 mg/h and 2.9 mg/h, respectively, after being immersed in the PaE solution (Supplementary Table S1). The difference depended on the proportion of the polymer that was easily degraded by the PaE.

### Degradation of BP films in a pipe house

**Figure 2:**
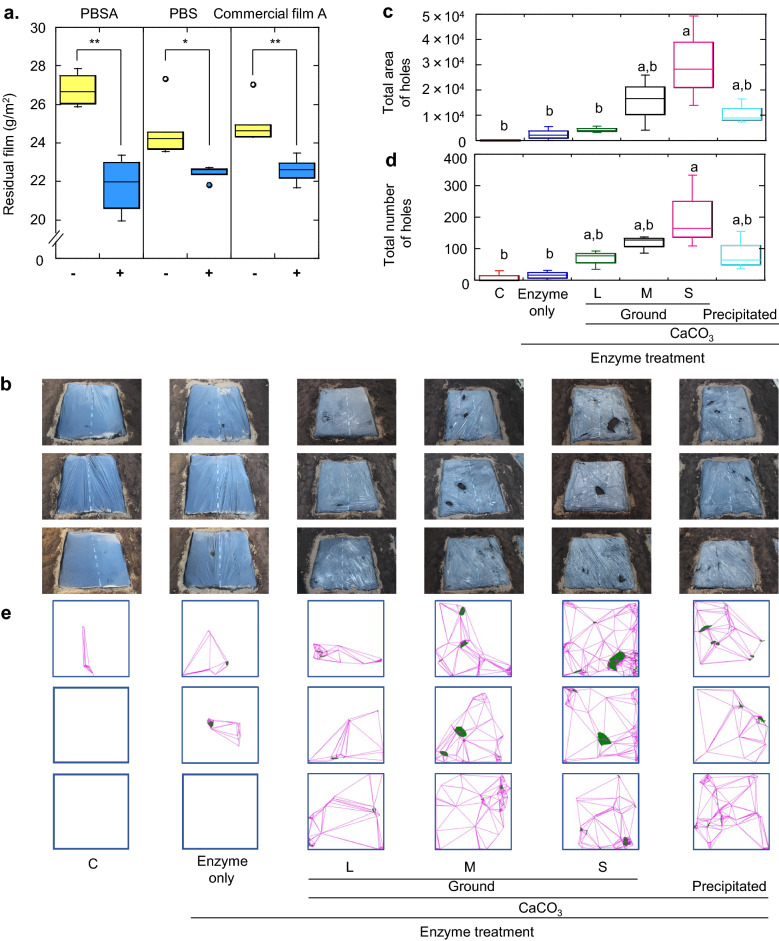
Enzyme treatment on BP films spread in a pipe house. (**a**) Weight reduction of various BP films. − : without treatment, + : enzyme treatment. Asterisks indicate that the data compared were significantly different (*n* = 6; *t*-test; ** *p* < 0.01, * *p* < 0.05). Effect of various calcium carbonates as enzyme stabilisers. (**b**) Images of the films the day after enzyme treatment, (**c**) total area of holes, and (**d**) number of holes in the films^§^. (**e**) Distribution of holes in the films. The holes are shown in green, and the distribution of the holes is shown by connecting the holes with magenta lines. C indicates without enzyme treatment, L, M, and S indicate treatment with heavy calcium carbonates of large, medium, and small particle sizes, respectively. “Precipitated” indicates treatment with precipitated calcium carbonate. (**c**) Values calculated based on the pixels of the images. Letters above multiple columns indicate that the values were significant (*p* < 0.05; *n* = 3) according to analysis of variance (ANOVA) using Tukey’s post-hoc test. ^§^We have been granted a patent for using PaE to accelerate the degradation of biodegradable mulch films laid in the field, for calcium carbonate to increase the effect, and for calcium carbonate to be more effective with smaller particle size^27^. The test examples in the specification include one each of the films shown in (**b**) treated with enzymes alone, enzymes, and large or small particle size calcium carbonate.

Next, we examined BP film degradation in soil in a pipe house. PBSA, PBS, and commercial mulch film A, which were prepared by blown extrusion, were spread on 1 m^2^ flat ridges, and the surfaces were treated with culture filtrate, hereafter referred to as enzyme solution (PaE 5.7 U, 400 mL/m^2^), using a pesticide applicator that all farmers already had. Although the water required for enzymatic hydrolysis was limited, a video of the PBSA film (video provided in Supplementary Information) showed that it decomposed until large holes (maximum diameter of 22 cm and an average of 9 cm) were opened before the film dried. Because the soil surface was not exactly horizontal, the surface of the film over the concavity soil was filled with enzyme solution, which reacted until the liquid dried or the film was perforated. The duration of hydrolysis of the film on convex soil was shorter due to the rapid drying of the surface. The weights of all enzyme-treated PBSA, PBS films, and film A recovered after 7 days (Fig. 2a) were significantly reduced (*t*-test, *p* < 0.01, *p* < 0.05, and *p* < 0.01, respectively), suggesting that the films were thinned by the enzyme treatment. The effect was confirmed by repeating the experiment using film A. The mean weight of the film after the treatment with PaE (4.3 U, 300 mL/m^2^) was 23.2 (STEV = 0.3) g/m^2^, and it was significantly different (*t*-test, *p* < 0.01) from the mean weight of untreated film, which was calculated as 23.9 (STEV = 0.47) g/m^2^.

### The effect of calcium carbonate on the PaE-induced degradation of BP films in the laboratory

During the degradation process of the polyester film, carboxylic acids are generated, and the reaction solution becomes acidic. Because the optimum pH for PaE is between 9.0 and 10.0 in Tris–HCl buffer^23^, calcium carbonate was selected to stabilize the pH when the ability of PaE to degraded polyester films was tested. Calcium carbonate is commonly used in the field as a neutraliser to reduce soil acidification and is therefore a suitable material for our study. Calcium carbonate is considered effective in mitigating the inhibition of enzyme reactions by insolubilizing organic acids produced by enzymatic degradation of the film. Commercial film A was treated with PaE and calcium carbonate in several laboratory experiments (Supplementary Information file, *S1*). In the immersion treatment of commercial film A (1.5 cm × 5 cm), the pH of the PaE reaction solution was maintained using 0.5–2% calcium carbonate (Supplementary Fig. S2a). Using more than 2% calcium carbonate significantly reduced the weight of the films (Supplementary Fig. S2b), as well as their tensile strength (Supplementary Fig. S2c), *p* < 0.01. Next, the film surface was covered with PaE to simulate an enzymatic treatment in the field. The treatment ratio of PaE 6 U at 200 mL/m^2^ in the field was reduced to a laboratory scale, and 0.24 mL of PaE (6 U) were applied to the surface of a piece of commercial film A (2.4 × 5 cm). Calcium carbonate (0.5 to 2.5%) was added to maintain the pH during the enzyme reaction (Supplementary Fig. S2d). It significantly increased the rate of film degradation area, *p* < 0.05 for > 1% calcium carbonate and *p* < 0.01 for more than 1.5% calcium carbonate (Supplementary Fig. S2e). The higher the concentration of calcium carbonate used in the experiment, the higher the pH after the reaction and the greater the amount of film degradation. On the other hand, the area of the film treated with calcium carbonate without PaE was unchanged (Supplementary Fig. S2e), indicating that calcium carbonate effectively stabilized the PaE activity, but it alone did not affect the degradation of the film.

Next, the ability of PaE to degrade films with different blends of PBAT (20, 40, 60, 80, and 100%) was evaluated in HEPES buffer, water, and calcium carbonate saturated solutions (Supplementary Information file, *S2*). Calcium carbonate is slightly soluble in water (6.8 mg/L at 25 °C)^28^. The film degradation activity in each solution was determined based on the increase in absorption values at 240 nm (A 240) which were caused by molecules containing aromatic rings that were eluted from the film into the reaction solution (Supplementary Information file, *S2*). The A 240 values after the reaction of PaE with all the films were significantly higher in the saturated calcium carbonate solution and in the HEPES buffer than in water. As the PBAT content of the film increased, so did the A 240 value after the reaction (Supplementary Fig. S3), indicating that the increase of the PBAT content of a film did not inhibit the PaE reaction. Phylloplane yeasts that produce enzymes similar to PaE can be isolated from most healthy leaves at low densities^22^. We further investigated whether the method could be applied to three enzymes from phylloplane eukaryotic microorganisms with previously reported BP-degrading activity, that is, PCLE from the ascomycete fungus *Paraphoma* B47-9^18^ and CfCLE from the basidiomycete yeast *Cryptococcus flavus* GB-1^19^, which degrade PBAT, and CmCut1 from the *Cryptococcus magnus*, which degrades various BPs but has not been investigated for PBAT degradation as yet^29,30^. Some of the enzyme-film reactions were more favorable in saturated calcium carbonate solutions than in water. And PaE showed the highest performance compared with the three fungal enzymes obtained from leaf surfaces (Supplementary Information file, *S2,* Supplementary Fig. S3).

### The effect of calcium carbonate on the PaE-induced degradation of BP films in a pipe house

The culture filtrate (PaE 3.4 U, 300 mL/m^2^) mixed with 1% of various kinds of calcium carbonate was sprayed on the surface of a 1 m square of film A on the ridge in a pipe house. In the next day's film surface images (Fig. 2b), the addition of calcium carbonate resulted in a significant increase (Tukey’s post-hoc test, *p* < 0.05) in the total area (Fig. 2c) and number (Fig. 2d) of holes, with the smallest particle size (SOFTON) being most effective. The distribution of the holes was shown by lines drawn to connect the holes on the film surface (Fig. 2e). The larger the total area and the higher the total number of holes, the more holes were distributed throughout the film, and such films were fragile.

### Degradation of BP films in open fields

**Figure 3:**
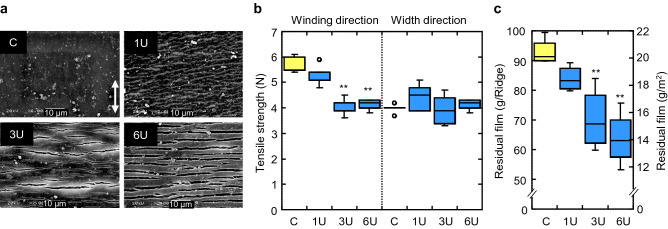
Effect of enzyme concentration on commercial BP mulch film A in an open field*.* (**a**) Micro morphology of film observed by SEM. The arrow indicates the winding direction of the film. (**b**) Tensile strength after enzyme treatment (*n* = 5). (**c**) Weight of residual films after ploughing the 5-m ridge; C: without enzyme treatment; 1 U, 3 U, and 6 U indicate enzyme concentration. C (without enzyme) was significant (** *p* < 0.01, Dunnett’s test). Each test was repeated independently (*n* = 4).

The water and warm temperature required for hydrolysis by enzymatic reaction are more easily maintained in a pipe house than in the field. However, as biodegradable mulch films are typically used in open fields, the effect of enzyme treatment on the film spread in an open field for one month was examined. As the submerging and coating treatment of PaE with more than 2% calcium carbonate (SOFTON) was significantly more effective in the laboratory experiment (*p* < 0.01, Supplementary Information file, Supplementary Fig. S2e), film A was treated with the enzyme solution (PaE 1 U, 3 U, and 6 U, 200 mL/m^2^) containing 2% SOFTON in the field at 20 °C (Supplementary Information file *Experiment 1,* Supplementary Table S2) and observed the next day via scanning electron microscopy (SEM) (Fig. 3a). Cracks were observed in the width direction; when the concentration of the enzyme was higher, the number and size of cracks increased, but no cracks were observed on the film without the enzyme treatment (Fig. 3a). The tensile strength of the films in the winding direction decreased at enzyme concentration higher than 3U (Dunnett’s test,* p* < 0.01, Fig. 3b), which was due to the cleavage of the polymer chains across the winding direction of the film by PaE. Such films are more likely to break into small fragments and easier to bury in soil using a tiller. The total weight of the remaining film after ploughing with a waking-type tiller decreased with enzyme concentration higher than 3U (Dunnett’s test,* p* < 0.01, Fig. 3c). When the amount of enzyme applied to film A was reduced to 120 mL/m^2^ (Supplementary Information file *Experiment 2*, Supplementary Table S2), a significant reduction in residual film fragments after ploughing was observed the day after the enzyme solution (PaE 6 U) was applied to the entire film surface in combination with 2% SOFTON. Partial treatment of the film surface had no effect on ploughing (Dunnett’s test*, p* < 0.05; Supplementary Fig. S4a).

**Figure 4:**
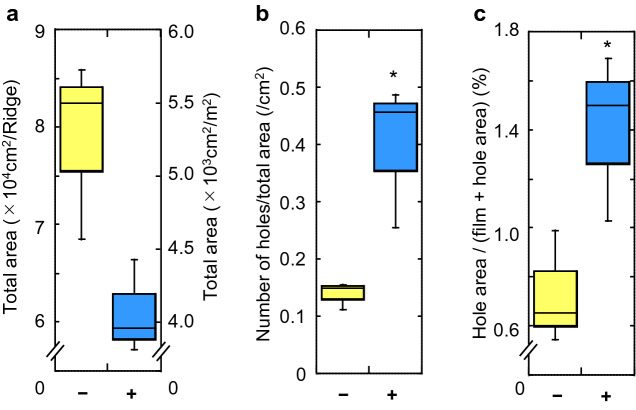
Effect of enzyme treatment on the residual film fragments collected from each ridge after ploughing. Commercial BP mulch film A on a 15-m ridge was treated with and without enzyme solution (PaE 6U and 2% SOFTON, 100 mL/m^2^). (**a**) Total area of film fragments collected, (**b**) number of holes per total area of collected fragments, and (**c**) ratio of the total area of the holes to the total area of the recovered fragments containing holes. *Asterisks indicate that the data compared are significantly different (*p* < 0.05, *t*-test). –: without enzyme treatment; + : with enzyme treatment.

Degradation of the durable mulch film B (which had a high PBAT) due to treatment with enzyme solution (PaE 6U with 2% SOFTON, 120 mL/m^2^) on the whole surface of the mulch film was also examined in late autumn (at 14 °C) during the two years of study (Supplementary Information file *Experiment 3, 4*, Supplementary Table S2). There was a reduction in the tensile strength of the film by the enzyme treatment (*t*-test*, p* < 0.01), and different ridges in the same field had almost the same reduction in film strength (Tukey’s post hoc test, *p* < 0.01, Supplementary Fig. S4b). The reduction in the total weight of remaining fragments on the ridge after the soil was ploughed using a waking tiller (*t*-test*, p* < 0.05, Supplementary Fig. S4c). The enzyme treatment was also effective in commercial BP mulch film C consisting of PBAT:PBSA:PBS:PLA at 26:42:22:10 (Supplementary Information file *Experiment 5,* Supplementary Table S2, Supplementary Fig. S4d, e). Therefore, treatment of 6 U PaE and 2% SOFTON applied to the entire film surface at 120 mL/m^2^ reproducibly reduced the strength of PBAT-based commercial mulch films used in early summer at the end of spring harvest (warm conditions) and at the end of autumn harvest (cold conditions) and made them more likely to be buried by ploughing (Supplementary Information file *Experiment 2–5*, Supplementary Table S2, Supplementary Fig. S4a-e). Assuming a more practical situation, mulch film A spread over a 15-m ridge was treated with enzyme solution (PaE 6U and 2% SOFTON, 100 mL/m^2^) at 27 °C, and it was ploughed using a tractor the next day (Supplementary Information file *Experiment 6,* Supplementary Table S2). This treatment significantly reduced tensile strength, total weight of remaining fragments (Dunnett’s test, *p* < 0.01, Supplementary Fig. S4f., g) as well as the total area of fragments (*t*-test*, p* < 0.05, Fig. 4a). On the other hand, the number of holes per total area of fragments collected was significantly increased (*t*-test, *p* < 0.05, Fig. 4b). The ratio of the total area of the holes to the total area of the recovered fragments that contained holes was also significantly increased (*t*-test*, p* < 0.05, Fig. 4c). In large film fragments of the enzyme treatment, the edges of the fragments appeared to be more complex, and many internal cleavages were observed (Supplementary Fig. S5a). The length of edges per area of each fragment (Supplementary Fig. S5b) and the total perimeter length of the holes per area of each fragment (Supplementary Fig. S5c) were greater in the enzyme-treated fragments than in the untreated fragments. These results show that the treatment reduced the strength of the film, which degraded into smaller fragments after ploughing.

## Discussion

In this study, we demonstrated that the leaf surface yeast esterase PaE could degrade biodegradable polyester PBAT films. In laboratory experiments, PaE randomly cleaved the interior of the PBAT polymer chain using an end-type attack. The resulting oligomers were scraped off the surface of the film, thus making the film thinner. In the field, commercial BP mulch films with different blends of PBAT were placed on the ridges, and their strength was reduced the day after PaE treatment in each independent experiment (Fig. 3b, Supplementary Fig. S4b, d, f). Cracks developed on the surface of the film, and the cracking increased as enzyme treatment concentrations increased (Fig. 3a). Based on the results of the laboratory experiments, PaE treatment of the film surface in the field also reduced the bulk of the high-molecular-weight polymer aggregates and thinned the films. The PaE pretreated films recovered from the soil after ploughing the following day were smaller in size but had longer edges (Supplementary Fig. S5b) and many larger holes inside (Fig. 4b, c, Supplementary Fig. S5c). Biodegradable films are more susceptible to surface erosion because the ratio of surface area to volume increases as the size and thickness of the fragments decrease^31,32^. The total weight (Fig. 3c, Supplementary Fig. S4a, c, e, g) and area (Fig. 4a) of the film recovered after ploughing also decreased. This was because the film fragments ploughed after the PaE treatment became too small to be visually recovered from the soil. In the six independent experiments conducted in this study, the effect on the total weight loss of the PaE-pretreated films recovered after ploughing was reproducible (Fig. 3c, Fig. 4a, c test 1 and 2, d, g). This effect was reproducible regardless of the type of film, including different amounts of PBAT and films with more durable PLA added, and regardless of the effects of early summer and late autumn temperatures. The results also showed that the PaE treatment increased the rate at which the film was incorporated into the soil. Because there were not as many pieces of film left on the field surface, its dispersion to the external environment was reduced.

Previously, laboratory experiments showed that commercial mulch film A pretreated with PaE lost its shape more quickly when it was buried in the soil^26^. In addition, changes in soil fungal flora occurred after the films were buried, and faster changes and recovery to the condition of original flora were observed in soil with enzyme-treated films^26^. This suggests that enzyme pre-treatment may accelerate the recovery of soil environments after ploughing the BP mulch film into field soils. In the future, it will be necessary to observe the degradation of the film in field soil and evaluate the effects of enzyme treatment in vegetable cultivation fields. The method of activating the degrading function of BPs by enzyme treatment allow the cultivation of a variety of vegetables with one durable BP mulch film. The method would be valuable for vegetable production if the total labor, cost, and environmental impact of post-use processing are comparable to or less than those of non-degradable mulch films.

In laboratory experiments, immersing PBAT films in a PaE solution at room temperature (25℃) caused the polymer chains to break (Fig. 1b, Supplementary Fig. S1), and induced erosion from the surface (Fig. 1a), leading to thinner films and reduced weight (Supplementary Table S1). In the field, the efficacy of the BP-hydrolyzing enzyme under drastic water and temperature conditions, thereby showing that enzymes can not only degrade mulch films but can also be used in a wide range of scenarios. Similarly, studies are underway to enzymatically degrade polyethylene terephthalate (PET) under optimal conditions, assuming the recovery of used polyesters ^20^. Although PET is classified as a plastic that does not decompose at ambient temperature, BP is degraded more efficiently by environmental microbial enzymes at ambient temperature. Using the BP products makes it more feasible to create a system that promotes degradation after the plastic is no longer needed^1^, as can be seen in this study. A variety of industrial enzymes are used because they act specifically and rapidly under mild conditions. Enzymes are proteins that are easily biodegraded and usually have low or no toxicity when released into the environment^33^. The safety of the enzyme solution (culture filtrate PaE 6 U) was confirmed by an outside agency to be negative for either bacterial reverse mutation activity, acute oral toxicity in rats and primary skin sensitization in humans (Supplementary Information File, Safety evaluation of enzyme solutions by external organizations).

Access to BP products and degrading enzymes is not an obstacle to the development of new ways to use plastics. Durable BP formulations that can be used in mulch films also have physical properties that can replace a variety of conventional nondegradable plastic products. The demand for alternatives, from conventional non-degradable plastics to durable BP products^7^ has increased the global PBAT production capacity by more than 280,000 tonnes in 2020. Of this, approximately 72,000 tonnes will be for agriculture and horticultural applications, and the remainder for packaging, coatings, and adhesives. The PBAT production capacity is expected to increase by approximately 40% by 2025^34^. In addition, we had previously developed methods for efficient production^35^ and stabilisation^36,37^ of PaE, which would enable the use of PaE on a large scale and at a low cost. Therefore, PaE may prevent the accumulation and inadvertent dispersal of BP, thereby making it easier to use in both open and closed environments.

## Methods

The biodegradable polymer resin pellets used were PBAT (Ecoflex F blend C1200, BASF SE, Ludwigshafen am Rhein, Germany), PBSA (BioPBS FZ91PB, PTT MCC Biochem Co., Ltd., Bangkok, Thailand), and PBS (BioPBS FD92PB, PTT MCC Biochem Co., Ltd.). The solution-cast film of PBAT was prepared in this study according to a previously described method^25^. PBAT, PBSA, and PBS heat-pressed sheets with a thickness of 50 μm were prepared by Mitsubishi Chemical Co., (Tokyo, Japan) by the following method: each pellet (1–2 g) was preheated at 240 °C for 5 min, pressed for 2 min, and then cooled by pressing at 25 °C for 5 min using a mini-test press machine MP-2 FH (Toyoseiki, Tokyo, Japan). The average molecular weights (Mn, number-average molecular weight; Mw, weight-average molecular weight; and Mw/Mn, polydispersity) of the polymers determined are shown in Supplementary Table S3.

PBSA (Bionolle 3001G, Showa Denko K. K., Tokyo, Japan) and PBS (Bionolle 1001G, Showa Denko K. K.) films used were black coloured and produced by blown extrusion, and they were 135 cm wide films. The typical values of average molecular weights were 20 to 25 × 10^4^ and that of thickness was 20 μm. Commercial BP mulch film A (16 μm thick, 23.0 g/m^2^) and B (14 μm thick, 23.3 g/m^2^) were composed of PBAT, PBSA, and PBS at a monomer-based weight ratio of 17:39:44 and 90:0:10, respectively. Similarly, the formulation of commercial BP mulch film C (20 μm thick, 28.3 g/m^2^) consists of PBAT:PBSA:PBS:PLA at 26:42:22:10. These compositions were estimated based on the molar ratio of monomers determined by liquid-state nuclear magnetic resonance (NMR), as described previously^38^ using Ecoflex F-Blend C1200 for PBAT and Bionolle 3001 and 1001 for PBSA and PBS, respectively, as reference materials. Of the constituents of each A, B, and C film, 97, 98 and 97% of the total weight were subjected to NMR analysis. These ratios were calculated from the dry weights obtained by dissolving each film (4 cm × 4 cm) in 750 μL of chloroform and then collecting the undissolved fractions using glass filters (GS -25 *ϕ*47 mm; Advantech, Tokyo, Japan).

The BP-degrading enzyme PaE was purified using the affinity method, as described previously^29^ to determine whether the PaE degrades the cast PBAT film. A 72-h xylose-fed batch culture filtrate using a jar fermenter of *P. antarctica* PaE-overexpressing strain XG8^35,36^ was used for the submerged treatment of BP sheets and films in PaE, and that of *P. antarctica* GB-4 (0) -HPM7 [MAFF 3070010]^39,40^ was used as an enzyme solution for film treatment in the field. The activity of PaE was evaluated based on the decrease in turbidity of emulsified PBSA (Bionolle EM-301, Showa Denko K. K.)^22^. One unit of PBSA degradation activity was defined as a 1-U decrease in absorbance at 660 nm/min in the reaction mixture at a 10-mm light path length at 30 °C in 20-mM Tris–HCl buffer at a pH of 6.8. The specific activity of the purified PaE was 20.02 U/mg (standard deviation:1.36).

### Molecular weight analysis

The average molecular weights of BPs were determined by size-exclusion chromatography (SEC). To determine the average molecular weights of PBAT resin pellets or PaE-treated cast PBAT films, samples dissolved in chloroform were applied to the Tosoh 8020 GPC system (Tosoh, Tokyo, Japan) equipped with two tandemly linked Shodex GPC LF-804 columns, a guard column Shodex LF-G, and a refractive index detector RI-8020 (Tosoh), as described previously^21^. The average molecular weights of heat-pressed sheets were measured by the Rhombic Corporation (Mie, Japan) as follows. Samples were applied to the Waters 2695 system (Milford, MA) equipped with two tandemly linked TSK Gel G5000H_HR_ and G3000H_HR_ columns (Tosoh), and a refractive index detector (2414, Waters). Chloroform was used as the eluent at 1.0 mL/min, and the column temperature was maintained at 40 ℃ during the analysis. To generate a calibration curve, commercial monodisperse polystyrene samples, listed in Supplementary Table S4, were used.

### LC–MS analysis of PBAT cast films after PaE treatment

The preparation of the solution-cast PBAT film, the treatment of the film with purified PaE, the LC–MS analysis of the reaction solution were all performed using previously described methods^25^.

### Degradation of sheets and films in vitro

Screw-cap glass bottles (Mighty Vial 110 mL No. 8, Maruem Co., Osaka, Japan) were filled with 50 mL of reaction solution (culture filtrate of strain XG8 at a final PaE concentration of 1.1 μM with 20 mM Tris–HCl pH 8.0 buffer). The sheets and films were cut into 3 × 3 cm pieces and prepared in triplicate. Each sheet or film was immersed in the reaction solution and shaken at 30 °C at 60 rpm. The incubation time was 3 h for PBAT and PBS sheets and commercial mulch film A and B and 1 h for the PBSA sheet. Then, the content was air-dried on filter paper and the weight loss was measured. The reaction time was adjusted so that the degradation rate of each film was in the range of 10–65%.

### BP film degradation in a pipe house

A pipe house (20 × 5.5 m) with a device that automatically opens when the temperature exceeds 30 °C was used in the experimental field at the National Institute for Agro-Environmental Sciences, NARO (36°02,458 N, 14°11,731 E).

PBSA, PBS, and commercial film A were installed in the pipe house by the Latin square method with 1-m square flat ridges on July 3, 2012. On July 5, 2012 at 16:30 (local time) at a temperature of 40 °C, enzyme solution (PaE 5.7 U, 400 mL/m^2^) was sprayed on the surface of each ridge using a pesticide sprayer (*n* = 3). A video recording of the PBSA-film process was made for 20 h until the morning of the next day after enzyme application (video is available in Supplementary Information). After seven days of enzyme treatment, the film was collected, cut into 80 × 80 cm fragments, washed, air-dried, and weighed. On August 1, 2012, commercial A films were installed in ridges of 1-m square in the pipe house. On August 2, 2012, at 10:00 at a temperature of 42 °C, the crude enzyme solution (PaE 4.3 U, 300 mL) was sprayed (*n* = 3). The film (1-m square) was collected the next day, washed, air-dried, and weighed.

### The effect of calcium carbonate on the PaE-induced degradation of BP films in the laboratory

Detailed information on the experimental methods and the results of are provided in *S1* and *S2* in the Supplementary Information File.

*S1* Selection of the calcium carbonate concentration suitable for enzymatic degradation of commercial biodegradable film.

*S2* Effect of the composition of the reaction solution, PBAT ratio of the film, and enzyme used for degradation of the film with various PBAT contents.

### The effect of calcium carbonate on the PaE-induced degradation of BP films in a pipe house

Commercial A films were placed on 1 × 1 m ridges in the pipe house by the Latin square method on September 11, 2013. On the next day, the crude enzyme solution (PaE 3.4 U, 120 mL/m^2^) with 1% of calcium carbonate was sprayed (*n* = 3). Heavy calcium carbonates with biaxial mean particle sizes of 3.72, 1.58, and 0.35 μm, (reagent grade CaCO_3_, Wako Pure Chemical Corporation, Osaka, Japan and CLEF-NON, SOFTON3200, Shiraishi Calcium Kaisha, Ltd., Osaka, Japan) and precipitated calcium carbonate (Biolaito 0.45 μm, ARIAKE Co., Ltd, Hyogo, Japan) were used. Comparisons were made with PaE without calcium carbonate and without any treatment^27^. The following day, the image of the film surface was photographed. The shape of the film’s image of 1-m square was a trapezoid; this was corrected by cutting 5 cm on both sides. The resultant 90-cm square was binarised using Photoshop (Adobe Inc., San Jose, California, USA), and the number of holes, total area of the holes, and distribution of the holes were analysed using WinROOF 2018 (MITANI Corporation, Tokyo, Japan).

### Degradation of BP films in open fields

Commercial BP mulch film A was placed on a flat ridge in the field for 29 days, and crude enzyme solutions (PaE 1, 3, and 6 U, 200 mL/m^2^) with 2% calcium carbonate (SOFTON) were spray treated over the film (*n* = 4) at a temperature of 20 °C (Supplementary Table S2, *Experiment 1*). A piece of film was collected the next day. The surface of the film fragment was coated with a gold layer in an ion sputter (Hitachi E-1010, Tokyo, Japan) and observed via SEM (JSM-5610LV, JEOL, Japan) at an accelerating voltage of 15 kV. Tensile strengths of the collected films (1.5 × 5 cm) in the winding direction and width direction of the film were measured using PTT-100 (Fuji Impulse Co. Ltd., Osaka, Japan) under the conditions of a chuck distance of 10 mm and a test speed of at 300 mm/min (*n* = 5 if not mentioned). This device evaluates the maximum seal strength in Newton (N), specified in EN 868–5 for the manufacturing pouches and reels. The tensile strength in the winding direction was used for subsequent evaluations. It is recommended that biodegradable mulch film be ploughed in as soon as it is no longer needed. Therefore, the film was ploughed under the day after the enzyme treatment. To determine the extent to which the film fragmented during ploughing operations, the soil in the ridges was ploughed twice using a waking-type tiller, and visible film fragments on the surface of the soil and up to a depth of 15 cm were collected, washed with water, and weighed after air-drying. Repeated experiments in the field were conducted to optimise enzyme treatment conditions, and each design is listed in Supplementary Table S2, and methods and results are presented in Supplementary Information File. *Experiments 2* was conducted to determine the effect of enzyme treatment method and calcium carbonate on film degradation. The effect of enzyme treatment on commercial mulch film B with higher PBAT content in cool weather conditions was examined in *Experiment 3, 4.* The film was placed on an 8-m ridge for one and a half months and treated with enzyme solution (PaE 6 U) containing 2% SOFTON (*n* = 3) at 14 °C in each experimental year. In the second year of the study, the same experiment was also done with film PLA added C (Supplementary Table S2, *Experiment 5*). Assuming a more practical situation, commercial film A was placed on a flat ridge of 15 × 1 m for 35 days, and PaE 3 or 6 U/mL with or without 2% SOFTON was sprayed over the entire surface of the film at 100 mL/m^2^ (*n* = 3) at 27 °C (Supplementary Table S2, *Experiment 6*). The next day, the soil was ploughed using a riding tractor, and the visible film fragments were collected, washed with water, and weighed after air-drying. Film fragments collected from each of the three ridges of the group without enzyme treatment and the 6-U/mL enzyme treated with SOFTON group were glued onto the surface of a piece of white paper (78.8 cm width) to read the image using a flatbed wide-format scanner (K-IS-A0FW, Array Co. Tokyo, Japan) and stored as digital data. After the acquired images were binarised, the areas and perimeters of each fragment and the holes generated in the fragment were measured using WinROOF 2018. The tensile strength in the winding direction of the enzyme-treated film was evaluated, as described above.

## Supplementary Information

Supplementary Video 1.

Supplementary Information 1.

## Data Availability

The datasets generated and/or analysed during the current study are available from the corresponding author on reasonable request.
