# Supplementary material for: Accelerated degradation of plastic products via yeast enzyme treatment
Source: Sci Rep. 2023 Feb 10;13:2386. doi: 10.1038/s41598-023-29414-1 (PMC9918467; doi:10.1038/s41598-023-29414-1)
Supplement: Supplementary file 2 — Supplementary Information 1. [file 41598_2023_29414_MOESM2_ESM.docx]

**Supplementary Information File**

**Accelerated degradation of plastic products via yeast enzyme treatment**

*Hiroko Kitamoto^1,*,#^, Motoo Koitabashi^1,#^, Yuka Sameshima-Yamashita^1,#^, Hirokazu Ueda^1^, Akihiko Takeuchi^1^, Takashi Watanabe^1,§^, Shun Sato^2^, Azusa Saika^2^, and Tokuma Fukuoka^2^*

*^1^National Agriculture and Food Research Organization (NARO), Tsukuba, Japan; ^2^Research Institute for Sustainable Chemistry, National Institute of Advanced Industrial Science and Technology (AIST), Tsukuba, Japan*

*^*^ Corresponding author*

*^#^ These authors contributed equally*

*^§^ Present address: Gunma Industrial Technology Center, 884-1 Kamesato, Maebashi, Gunma 379-2147, Japan*

**SUPPLEMENTARY INFORMATION**

**MATERIALS AND METHODS**

**LC-MS analysis of PBAT cast films after PaE treatment**

The preparation of the solution-cast PBAT film, the treatment of the film with purified PaE, the LC-MS analysis of the reaction solution of the residual film were all performed using previously described methods^1^.

**■The effect of calcium carbonate on the PaE-induced degradation of BP films in the laboratory**

***S1*. Selection of the calcium carbonate concentration suitable for enzymatic degradation of commercial biodegradable film**

Preparation of the submerge treatment involved the following steps: the submerged treatment consisted of screw-cap glass bottles (Mighty Vial 110 mL No. 8, Maruem Co., Osaka, Japan) filled with 10 mL of water containing enzyme solution (1U PaE) with 0, 0.5, 1, and 2% calcium carbonate (SOFTON). Commercial BP mulch film A cut to 1.5 cm × 5 cm was immersed in the reaction solution and shaken for 30 min at 30°C with an amplitude of 25 mm at 130 rpm using a reciprocal shaker (NR-3, Taitec Saitama, Japan) (*n* = 3). After the film was rinsed with water and dried, the weight reduction, tensile strength and the pH of the reaction solution were evaluated. Films treated with water were used for comparison. Tensile strengths of the films were measured using PTT-100 (Fuji Impulse Co. Ltd., Osaka, Japan) under the conditions of a chuck distance of 10 mm and a test speed of at 300 mm/min (*n* = 5 if not mentioned).

Preparation of the coating treatment involved the following steps: the BP mulch film A (2.4 × 5 cm) was treated with the enzyme solution (PaE 6 U, 240 μL) with 0, 0.5, 1, 1.5, 2, and 2.5% SOFTON in a glass Petri dish with a lid (*n* = 3) in the same manner as in the open field experiment for enzyme solution treatment (PaE 6 U, 200 mL/m^2^). To maintain humidity in the Petri dish, the film was covered with glass of the same size, and wet filter paper was placed in the dish and left at 30°C for 24 h. The film was then washed with water, dried, and photographed using a scanner. The reduction rate of the black film area was calculated using the image-processing software Aquacosmos 2.0 (Hamamatsu Photonics, Shizuoka, Japan).

***S2*.** **Effect of the composition of the reaction solution, PBAT ratio of the film, and the enzyme used for degradation of the film with various PBAT contents**

The PBAT cast film was prepared as follows: an aliquot (5 mL) of a 4% solution of PBAT in chloroform, prepared from resin pellets (Ecoflex F-Blend C1200), was poured into a 6 cm-diameter glass Petri dish and heated to 55°C to evaporate the chloroform. The films were rinsed with water and dried overnight, thereafter, each film of 70–80 μm (7–8 mg/cm^2^) thickness was obtained. Additionally, 18-µm-thick films with different PBAT blending ratios prepared using a laboratory inflation molding machine were purchased from Shin-Etsu Polymer Co., Ltd. (Tokyo, Japan). The ratio of PBSA (Bionole 3001 MD):PBS (Bionole 1001 MD):PBAT (Ecoflex F Blend C 1200) per weight in each film was 40:40:20 (No. 1), 20:40:40 (No. 2), and 10:10:80 (No. 3).

Film samples (1 cm^2^ area), reaction solution (50 mM HEPES buffer (pH 7.3), Dojindo Laboratories, Kumamoto, Japan), water, and supernatant of a saturated calcium carbonate solution were prepared. The BP-degrading enzymes PaE^2^, PCLE from the ascomycete fungus *Paraphoma* B47-9^3^, CfCLE from the basidiomycete yeast *Cryptococcus flavus* GB-1^4^, and CmCut1 from the *Cryptococcus magnus*^5, 6^ were purified from each culture supernatant as described previously^5^. A quartz cuvette with an optical path length of 1 cm was filled with 2 mL of each reaction solution containing 1 nM purified enzyme solution. The film was then added and allowed to stand for 4 h at approximately 25°C with constant stirring. Then, the Absorbance_240_ due to the aromatic ring of terephthalic acid released into the reaction solution was measured using an ultraviolet-visible spectrophotometer V -530 ST (JUSCO Co., Ltd., Minato Ward, Tokyo, Japan). Three to seven measurements were taken for each condition. The mean value, excluding the maximum and minimum values of the results measured without the enzyme, was used as a blank, and the blank value was subtracted from each measurement.

**■Degradation of BP films in open fields**

The effect of enzyme treatment on commercial BP mulch films spread over ridges in an open field was examined for one month. To reduce the cost of the enzyme treatment, the amount of enzyme was reduced step-by-step by conducting repeated experiments (Table S2). The ridges that were randomly placed in the field were treated with the enzyme solution. The following day, the film was plowed using a waking-type tiller (*Experiment 1-5*) or riding tractor (*Experiment 6*) for two rounds. Visible film fragments on the surface of the soil up to a depth of 15 cm were collected, washed with water, and weighed after air-drying. The tensile strength in the winding direction of the enzyme-treated film was evaluated using PTT-100, as described above.

***Experiment 1*. Effect of enzyme concentration on the degradation of field-spread film**

Commercial BP mulch film A was placed in a 5 × 1-m flat ridge in the field on September 8, 2014, and 29 days later, on October 7, 2014, at a temperature of 20°C. Crude enzyme solutions (PaE 1, 3, and 6 U, 200 mL/m^2^) with 2% calcium carbonate (SOFTON) were sprayed onto the films (*n* = 4). A piece of the film at the margin of the ridge was collected the following day. The surface of the film fragment was coated with a gold layer using an ion sputter (Hitachi E-1010, Tokyo, Japan) and observed using SEM (JSM-5610LV, JEOL, Japan) at an accelerating voltage of 15 kV. Tensile strengths of the collected films (1.5 × 5 cm) in the winding direction and width direction of the film were measured. Two rounds of plowing of the soil in the ridges were conducted using a waking-type tiller. Visible film fragments on the surface of the soil up to a depth of 15 cm were collected, washed with water, and weighed after air drying.

***Experiment 2*. Effect of enzyme treatment method and calcium carbonate on film degradation**

Commercial BP mulch film A was placed on a 6 × 1-m flat ridge on June 19, 2015, and 32 days later, on July 21, 2015, at 31°C. PaE (6 U/mL) was sprayed onto the film surface, with or without 2% SOFTON. The four ridges that were randomly placed in the field were treated with the enzyme solution using the following three methods:1) the entire surface of the ridge was sprayed at 120 mL/m^2^; 2) the ridge was compartmentalized with 50-cm widths and PaE was applied at 1-compartment intervals (total 60 mL/m^2^); and 3) the ridge was alternately compartmentalized with widths of 40 cm and 10 cm, with only the 10-cm wide region treated with enzyme (total 24 mL/m^2^). The following day, on July 22, 2015, the film was plowed using a waking-type tiller for two rounds. Visible film fragments on the surface of the soil up to a depth of 15 cm were collected, washed with water, and weighed after air-drying.

***Experiments 3* and *4*. Effect of enzyme treatment at low temperature on degradation of the film with high PBAT content**

Commercial film B was placed on a flat ridge measuring 8 × 1 m. In *Experiment 3*, the film was spread on September 1, 2016, and 54 days later on October 25, 2016, at 14°C. PaE 5.6 U/mL with 2% SOFTON was sprayed over the entire surface of the film of the three ridges at 120 mL/m^2^. The next day (October 26, 2016), the soil was plowed with a waking-type tiller for two rounds, and the film fragments exposed on the soil surface were collected, washed with water, and weighed after air drying. Similarly, in *Experiment 4*, the film was spread on September 26, 2017, and 48 days later, on November 13, 2017, at 14°C. PaE of 6 U/mL with 2% SOFTON was sprayed over the entire surface of the film of the three ridges at 120 mL/m^2^. The next day (November 14, 2017), the soil was plowed with a waking-type tiller for two rounds, and the film fragments exposed on the soil surface were collected, washed with water, and weighed after air drying. The tensile strength in the winding direction of the enzyme-treated film was evaluated, as described above.

***Experiments 5*. Effect of enzyme treatment at low temperature on degradation of PLA-containing films**

The commercial BP mulch film C was cut into 3 × 3 cm pieces and prepared in triplicate. Each film was immersed in the reaction solution (50 ml of culture filtrate of strain XG8 at a final PaE concentration of 1.1 μM with 20 mM Tris-HCl pH 8.0 buffer) in a screw-cap glass bottles (Mighty Vial 110 mL No. 8, Maruem Co., Osaka, Japan) and shaken at 30 °C at 60 rpm, 3 h. Then, the content was air-dried on filter paper and the weight loss was measured.

On September 26, 2017, commercial BP mulch film C was placed on an 8 × 1-m flat ridge and enzyme treatment, ploughing and evaluation were conducted simultaneously with *Experiment 4*.

***Experiment 6*. Effect of enzyme treatment on ploughing commercial BP mulch film in soil using a riding tractor in the open field**

Commercial film A was placed on a flat ridge of 15 m × 1 m on August 24, 2015, and 35 days later, on September 28, 2015, at 27°C. PaE of 3 or 6 U/mL with or without 2% SOFTON was sprayed over the entire surface of the film at 100 mL/m^2^ (*n* = 3). Four ridges were randomly placed in the field. One day later, on September 29, 2015, soil was plowed using a riding tractor. Visible film fragments on the surface of the soil up to a depth of 15 cm were collected, washed with water, and weighed after air drying. Film fragments collected from each of the three ridges of the group without enzyme treatment and the 6-U/mL enzyme treated with SOFTON group were glued onto the surface of a piece of white paper (78.8 cm width) to read the image using a flatbed wide-format scanner (K-IS-A0FW, Array Co. Tokyo, Japan) and stored as digital data. After the acquired images were binarized, the areas and perimeters of each fragment and holes generated in the fragment were measured using WinROOF 2018. The tensile strength in the winding direction of the enzyme-treated film was evaluated, as described above.

**RESULTS**

**■LC-MS analysis of PBAT cast films after PaE treatment**

Various oligomers formed by polymer chain scission were eluted in the enzyme reaction solution, and the PBAT monomer butanediol was detected in the positive ion chromatograms [TIC(+)] (Supplementary Fig. S1).

**■The effect of calcium carbonate on the PaE-induced degradation of BP films in the laboratory**

Generation of carboxylic acids during the degradation of polyester films resulted in acidification of the reaction solution. As alkaline conditions are conducive to PaE activity^7^, the use of calcium carbonate, which is available in farmlands as a pH stabilizer, was tested in laboratory experiments for its ability to maintain enzyme activity.

***S1*. Selection of the calcium carbonate concentration suitable for enzymatic degradation of commercial biodegradable film**

For the submerge treatment in the laboratory, commercial film A (1.5 cm × 5 cm) was submerged in 10 mL of water containing 1 U of PaE for 30 min. The pH of the reaction solution decreased from 6.8 to 5 after the immersion. The addition of 0.5, 1, and 2% calcium carbonate (SOFTON) maintained the pH of the reaction solution at 7.1, 7.4, and 7.3, respectively (Supplementary Fig. S2a). The weight of the film was significantly decreased (*p* < 0.01) by 2% SOFTON treatment compared with PaE treatment alone (Supplementary Fig. S2b). The tensile strength of the film in the winding direction was further significantly decreased by 1% SOFTON (*p* < 0.05) or 2% (*p* < 0.01) treatment compared to the PaE treatment alone (Supplementary Fig. S2c).

Coating treatment: the amount of enzyme treatment per area in the laboratory experiment was maintained at the same level as in the open field experiment (PaE 6U, 200 mL/m^2^). Therefore, film A (2.4 cm × 5 cm) was treated with 0.24 mL of PaE (6 U) and SOFTON (0.5% to 2.5%) and allowed to stand for 24 h at 30°C. The higher the concentration of SOFTON, the higher the pH after the reaction (Supplementary Fig. S2d) and the better the degradation of the film based on the residual area of the film (Supplementary Fig. S2e). The area of the film treated with SOFTON without enzyme treatment was unchanged, indicating that SOFTON effectively stabilized the PaE activity, but SOFTON alone did not affect the degradation of the film (Supplementary Fig. S2e).

***S2*. Effect of the composition of the reaction solution, PBAT ratio of the film, and the enzyme used for degradation of the film with various PBAT contents**

The PBAT cast film and laboratory-made inflation films with 20–80% PBAT were immersed in a reaction solution containing PaE at 30°C for 4 h. An increase in the 240 nm absorption due to the aromatic ring of terephthalic acid released in the reaction solution was observed (Supplementary Fig. S3). The Absorbance_240_ in HEPES buffer and saturated calcium carbonate solutions was greater than that in water with PaE (Supplementary Fig. S3a–d). In the experiment using films with 20–80% PBAT immersed in HEPES and saturated calcium carbonate solutions containing PaE, the Absorbance_240_ increased with increasing PBAT content ratio of the films (Supplementary Fig. S3b, c, d). These results show that calcium carbonate maintained PaE activity during the degradation of the films in the submerged treatment of the 100% PBAT cast film, as well as in the inflation films blended with up to 80% PBAT, regardless of the percentage of PBAT. Yeasts that produce enzymes similar to PaE can be isolated from most healthy leaves uninfected by plant pathogens at low densities^2^. PaE exhibited the highest performance compared to that of three fungal enzymes obtained from leaf surfaces (PCLE^3^, CfCLE^4^, and CmCut1^5, 6^). PaE showed the highest performance compared with the three fungal enzymes obtained from leaf surfaces. Some of the enzyme-film reactions were more favorable in saturated calcium carbonate solutions than in water (Supplementary Fig. S3). In films with 100% PBAT, HEPES and calcium carbonate solutions promoted degradation by CmCut1 more than water (Supplementary Fig. S3a) ; in films with low PBAT content (20%), HEPES and calcium carbonate solution promoted degradation by CmCut1 and CfCLE more than water (Supplementary Fig. S3b). And in films with high PBAT content (40% and 80%), the HEPES buffer increased the degradation by the three enzymes compared to those in calcium carbonate solution and water (Supplementary Fig. 3c, d).

**■Degradation of BP films in open fields**

***Experiment 1*. Effect of enzyme concentration on the degradation of field-spread film**

Mulch film A was spray-treated with enzyme solutions (PaE 1, 3, and 6 U, 200 mL/m^2^) along with 2% calcium carbonate (SOFTON) at 20°C. The film was collected on the day after the treatment, and its surface was observed using SEM. Cracks were observed perpendicular to the winding direction of all enzyme-treated films; when the enzyme concentration was higher, the number and size of the cracks increased (Fig. 3a). No cracks were observed in the films without the enzyme treatment. The tensile strength of the films (1.5 × 5 cm) in the winding direction decreased with increasing enzyme concentration because the polymer chains cleaved across the winding direction of the film by PaE. When tensile force was applied in the width direction, no differences were observed among the various enzyme treatments (Fig. 3b). After treatment with the enzyme solution, micro- (Fig. 3a) and visible-sized (Fig. 2b) cracks were observed on the surface of the commercial films spread in the field. These results suggest that such films are more likely to break into small fragments when plowed using a tiller. The total weight of the film fragments that remained in the field after plowing the 5-m ridges with a waking-type tiller decreased based on the enzyme concentration. The median decreases were 9% at 1 U, 25% at 3 U, and 31% at 6 U, compared with that of the no enzyme treatment (control) (Fig. 3c).

***Experiment 2*.** **Effect of enzyme treatment method and calcium carbonate on film degradation**

When a reduced quantity of enzyme solution (PaE 6 U, 120 mL/m^2^) with 2% SOFTON was applied to film A on the 6-m ridge, the total weight of the film fragments that remained after plowing was significantly decreased by a median of 27% when compared with the control (*p* < 0.05, Supplementary Fig. S4a). When the enzyme solution was treated without SOFTON, the amount of film remaining decreased by 17%; however, the difference from the untreated enzyme treatment was not significant. In addition, spraying the crude PaE solution at doses of half (60 mL/m^2^) or one-fifth of the total area (24 mL/m^2^), with and without SOFTON, did not reduce the total amount of residual fragments after plowing (Supplementary Fig. S4a). Therefore, spray treatment of the enzyme solution with SOFTON over the entire surface of the film is necessary to reduce the mass of residual fragments after plowing.

***Experiments 3* and *4*. Effect of enzyme treatment at low temperature on the degradation of film with high PBAT content**

Many BP mulch films currently used in Japan are composed of more than 50% PBAT (data not shown). They have high durability and slow decomposition characteristics, and their desired covering functions have been improved during their service lives in various applications. In addition, degradation of the used film is necessary both in the warm early summer at the end of the spring harvest and in the cold season at the end of the autumn harvest. PaE activity during the degradation of the PBSA emulsion was found to be highest at 40°C^7^, suggesting that enzyme treatment under cool weather conditions decreases the degradation of the film in the field. Therefore, the effect of enzyme treatment on commercial mulch film B with a higher PBAT content under cool weather conditions was examined*.*

The film was placed on an 8-m ridge for one and a half months and treated with the enzyme solution (PaE 6 U) containing 2% SOFTON at 14°C in each experimental year. The tensile strength (*p* < 0.01, Supplementary Fig. S4b) and weight of the post-plowing residual films (34% and 19%, respectively) (*p* < 0.05, Supplementary Fig. S4c) significantly decreased after enzyme treatment in both years. These results indicated that spraying the enzyme solution (PaE 6 U) containing 2% SOFTON over the surface of the BP film containing PBAT was highly effective in reducing the total weight of the residual film fragments after plowing.

***Experiment 5*. Effect of enzyme treatment at low temperatures on the degradation of PLA-containing films**

To further increase the durability of biodegradable mulch films, those with added PLA were also used. As PLA decomposes very slowly in a normal-temperature environment, the proportion of PLA in commercial mulch film is small. Another advantage of PLA is that it is a relatively inexpensive plant-derived material. On the other hand, PaE does not degrade crystallized PLA, but slowly degrades its amorphous region^8^. In a laboratory experiment, a film of commercial biodegradable mulch C consisting of PBAT:PBSA:PBS:PLA at 26:42:22:10 was immersed in a solution of PaE, and, 4.0 mg of solids per hour was reduced. Film C was then spread and the enzyme was treated in the same manner as in *Experiment 4*. Results showed that enzyme treatment significantly reduced the strength of the film (*p* < 0.01) and the total weight of fragments recovered after ploughing (*p* < 0.05). The median total weight of fragments was reduced by 19% compared with that of no enzyme treatment (Supplementary Fig. S4d, e). Therefore, treatment of 6 U PaE and 2% SOFTON applied to the entire film surface at 120 ml/m^2^ reproducibly accelerated the degradation of PBAT-based commercial mulch films tested in early summer at the end of spring harvest (warm conditions) and at the end of autumn harvest (cold conditions). (Supplementary Fig. S4a-e).

***Experiment 6*. Effect of enzyme treatment on plowing commercial BP mulch film in soil using a riding tractor in the open field**

Assuming a more practical situation, mulch film A spread over the 15-m ridge was treated with PaE 100 mL/m^2^ with 2% SOFTON at 27°C. It was plowed using a riding tractor the following day. Treatment with the enzyme (3 U, 6 U) alone and with SOFTON decreased the tensile strength and amount of residual film. The reproducibility of both evaluations was high in the treatment with 6 U enzyme with SOFTON (*p* < 0.01, Supplementary Fig. S4f, g). Enzyme treatment (6 U with SOFTON) significantly reduced the total area of the fragments (Fig 4a), and the number of holes to the area of each fragment (Fig. 4b) and the ratio of the total area of holes to the total area of recovered fragments containing holes (Fig. 4c) were significantly larger than those in the untreated group (*p* < 0.05). In the large film fragments of the enzyme treatment, the edges of the fragments appeared to be more complex and many internal cleavages were observed (Supplementary Fig. S5a). Measurements showed that the length of the edges per unit area of each fragment (Supplementary Fig. S5b) and the total perimeter length of the holes per unit area of each fragment (Supplementary Fig. S5c) were greater in the enzyme-treated fragments than in the untreated fragments. These results indicated that the film on the ridge reduced the strength of the film after enzyme treatment, and the fragments became smaller after plouging.


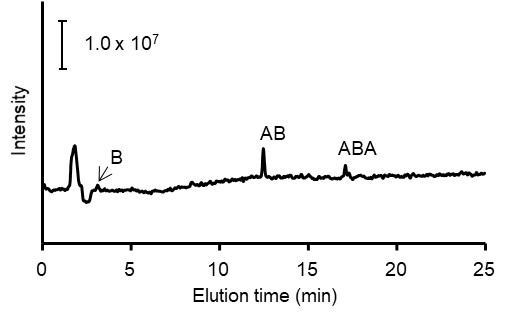


**Supplementary Fig. S1.** Total ion current chromatogram (+) of the water-soluble products of PaE-catalyzed degradation of PBAT film for 4 h, examined using liquid chromatography-mass spectrometry. A, adipate; B, butanediol; T, terephthalate.


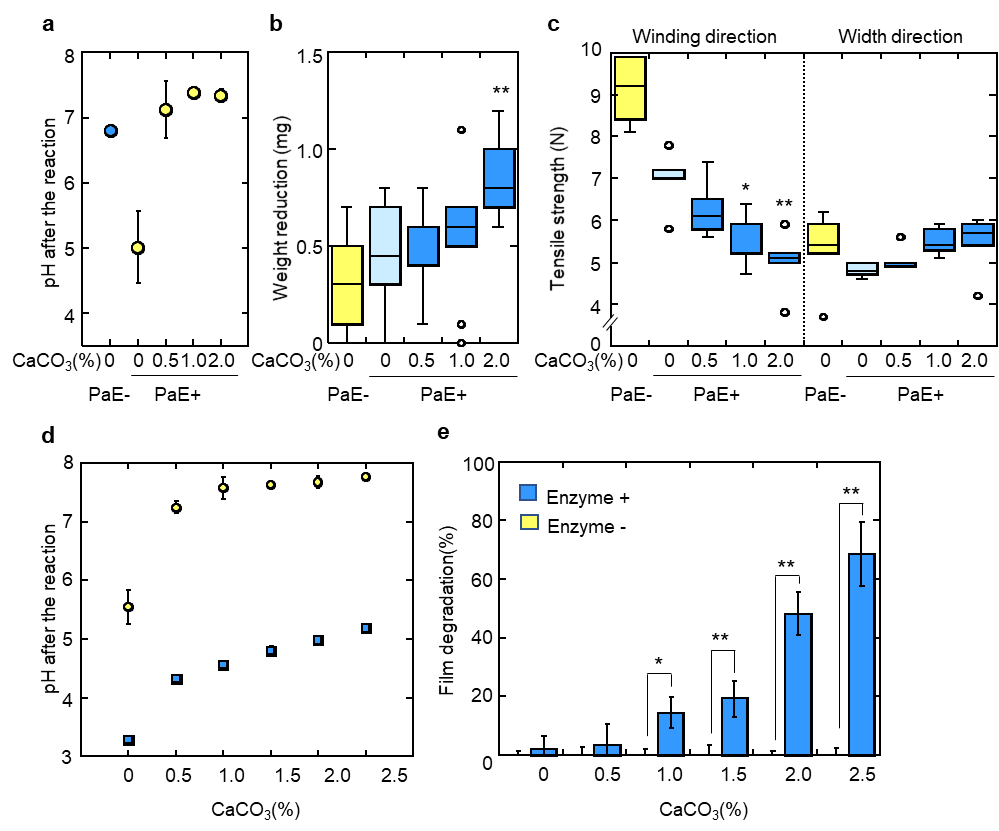


**Supplementary Fig. S2.** Effect of calcium carbonate concentration on the enzymatic degradation of commercial film A via PaE treatment.

**a.** pH of the reaction solution **b.** weight reduction of the film (1.5 cm × 5 cm) and **c.** tensile strength of the film by submerging treatment. SOFTON was used as the calcium carbonate (*n* = 3).

There was a significant difference between the results of PaE treatment with and without calcium carbonate (**p* < 0.05, ***p* < 0.01, Dunnett’s test).

**d.** pH of the reaction solution **e.** ratio of the film residual area and after coating treatment. SOFTON was used as calcium carbonate (*n* = 3). There was a significant difference between the values after arcsine square root transformation, with and without enzyme treatment (**p* < 0.05, ***p* < 0.01, Student’s *t*-test).

Yellow bars and circles indicate results without enzyme treatment, whereas blue bars and squares indicate results of enzyme treatment.

**
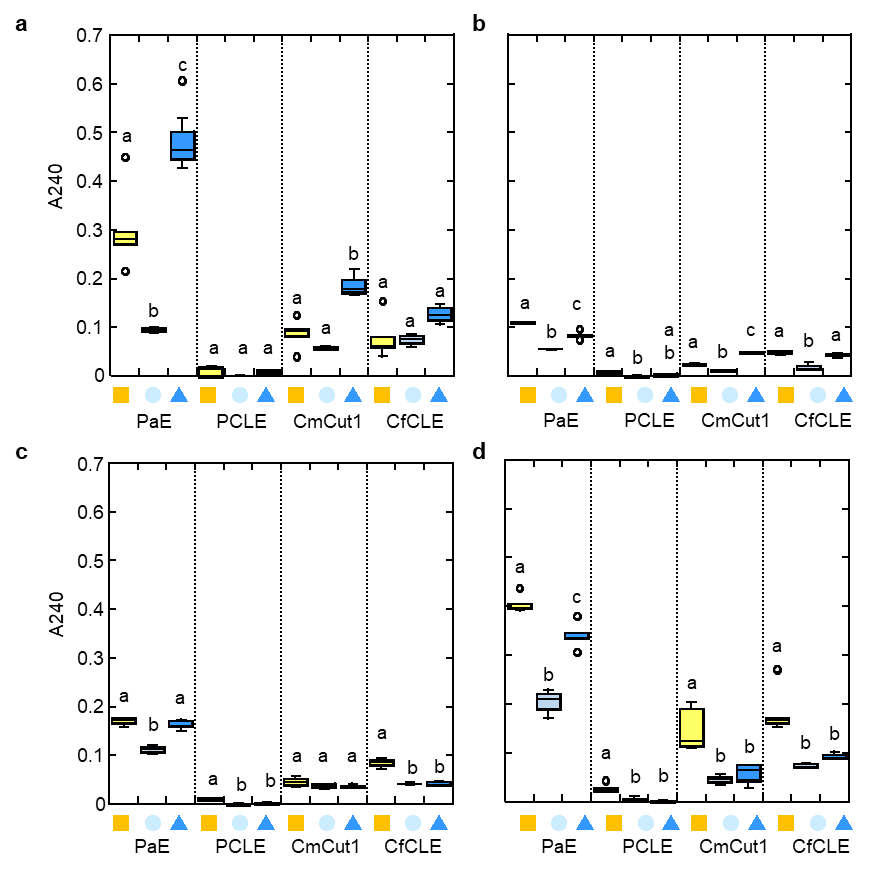
**

**Supplementary Fig. S3.** Effect of the composition of the reaction solution, PBAT ratio of the film, and the enzyme used for the degradation of the film with various PBAT contents

**a.** PBAT cast film, **b.** inflation film No. 1 containing 20% PBAT, **c.** inflation film No. 2 containing 40% PBAT, **d.** inflation film No. 3 containing 80% PBAT. The UV absorption of the aromatic compounds in the reaction solution measured at 240 nm. The value without the enzyme treatment was subtracted from the total value.

The reaction solutions used were HEPES buffer (orange squares), water (light blue circles), and a calcium carbonate saturated solution (blue triangles). Analysis of variance using Tukey’s post hoc test for each frame; letters in each box plot indicate significance (*p* < 0.05, *n* = 3–7).


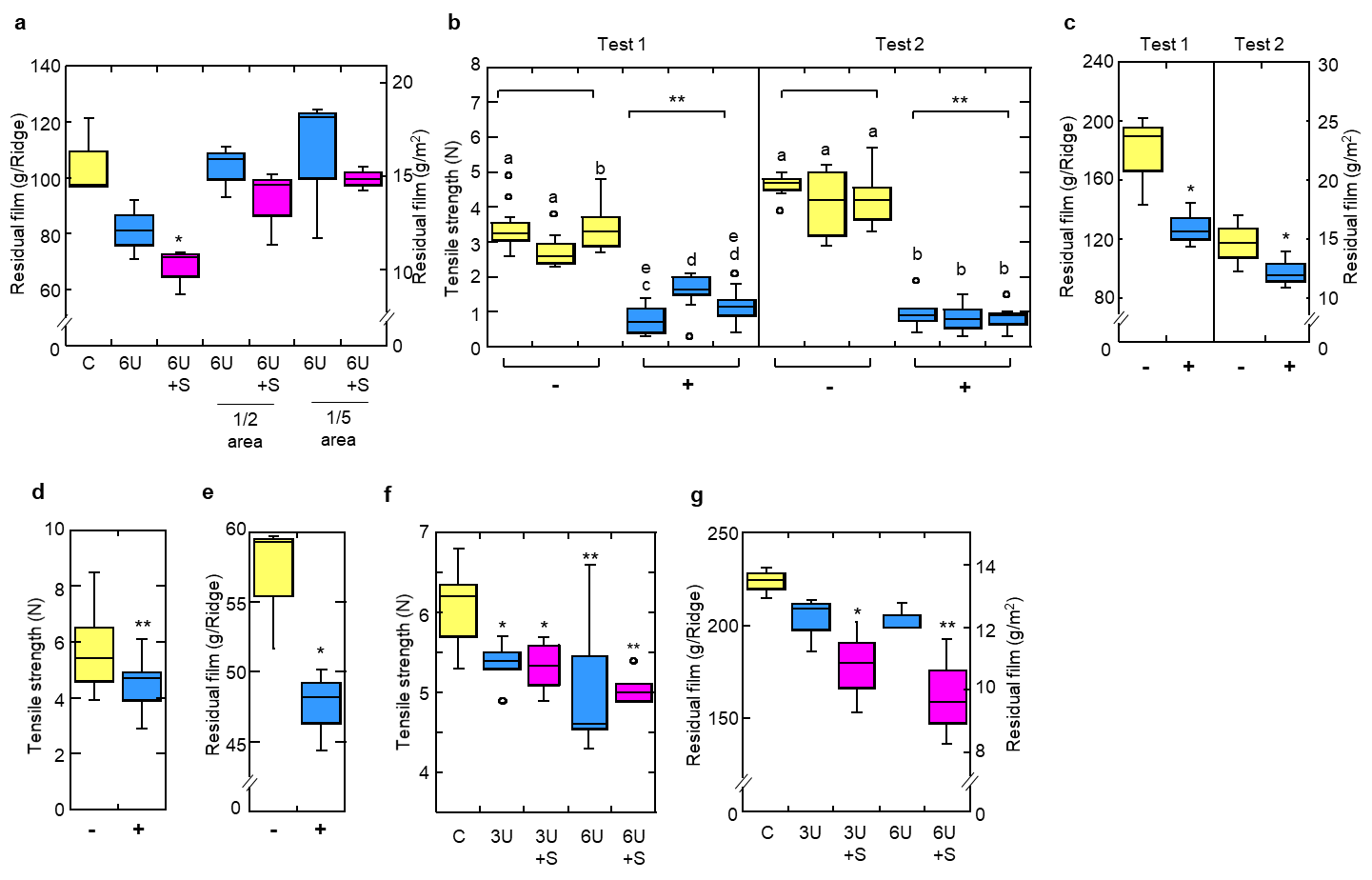


**Supplementary Fig. S4.** Effect of enzyme treatment conditions on promoting the degradation of commercial biodegradable plastic film in an open field.

**a.** Weight of residual BP mulch film A after ploughing 6-m ridges (*Experiment 2*). C: without enzyme treatment, 3 U and 6 U: concentration of treated enzyme solution, S: combination with 2% SOFTON. Asterisks indicate significant differences in data between the enzyme treatment and C (without enzyme treatment) (*p* < 0.05, Dunnett’s test). Each test was independently repeated (*n* = 3 each).

**b.** Tensile strength for each ridge and **c.** residual weight of BP mulch film B with high PBAT content after ploughing 8-m ridges in the late autumn of two years in an open field (Experiment *3, 4*), –: without treatment, +: enzyme treatment. Tukey’s post hoc test for each frame; letters in each box plot indicate significance (*p* < 0.01, n = 7–21). Asterisks indicate that the compared data were significantly different (** *p* < 0.01, * *p* < 0.05, *t*-test). Each test was independently repeated (*n* = 3 each).

**d.** Tensile strength and **e.** residual weight of PLA added BP mulch film C in each treatment after ploughing 8-m ridges in the late autumn in an open field (*Experiment* *5*), –: without treatment, +: enzyme treatment. Asterisks indicate that the compared data were significantly different (** *p* < 0.01, * *p* < 0.05, *t*-test). Each test was independently repeated (*n* = 3 each).

**f.** Tensile strength and **g.** weight of residual BP mulch film A after ploughing 15 m ridges with a riding tractor (*Experiment* *6*). C: without enzyme treatment, 3 U and 6 U: concentrations of the treated enzyme solution, S: combination with 2% SOFTON.

Asterisks indicate significant differences between enzyme treatment and C (without enzyme treatment) (** *p* < 0.01, *p* < 0.05, Dunnett’s test). Each test was repeated independently (*n* = 7; *n* = 3).


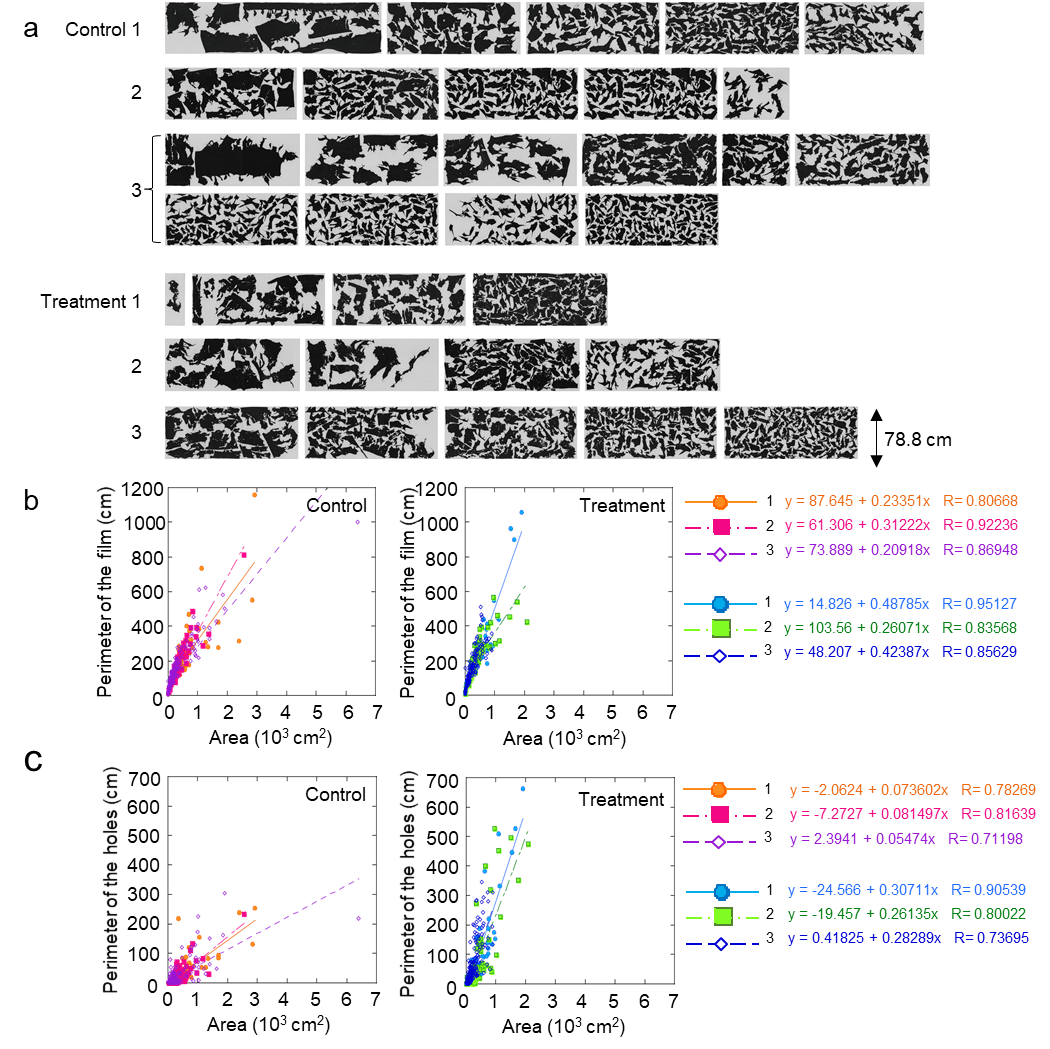


**Supplementary Fig. S5.** Shapes of the film fragments collected from the field after ploughing.

**a.** Shapes of the film fragments, **b.** area and perimeter of the film fragments, and **c.** area and perimeter of holes in the film fragments collected from the field after ploughing. Commercial BP mulch film A on 15-m ridges was treated with and without enzyme solution (PaE 6 U) and 2% SOFTON. Film fragments >10 cm^2^ are presented in **b** and **c** by enzyme treatment and untreated ridge, respectively*.* Control: without enzyme treatment, Treatment: enzyme treatment.

**Supplementary Table S1.** Weight reduction of neat heat-pressed films and commercial BP mulch films (3 × 3 cm) using the PaE submerging treatment

| Material | Weight before  reaction (mg) | | Weight reduction  (mg) | | Reaction time (h) | Degradation rate  (mg/h) | |
| --- | --- | --- | --- | --- | --- | --- | --- |
|  | Average | SD | Average | SD |  | Average | SD |
| PBAT | 59.1 | 0.4 | 7.0 | 0.5 | 3 | 2.3 | 0.18 |
| PBSA | 79.9 | 6.9 | 45.2 | 0.1 | 1 | 45.2 | 0.12 |
| PBS | 62.3 | 3.7 | 27.9 | 0.2 | 3 | 9.3 | 0.08 |
| Film A | 23.3 | 0.9 | 14.0 | 0.3 | 3 | 4.7 | 0.10 |
| Film B | 21.1 | 0.7 | 8.8 | 0.8 | 3 | 2.9 | 0.26 |
| Film C | 17.8 | 0.8 | 11.9 | 0.7 | 3 | 4.0 | 0.22 |

**Supplementary Table S2.** Field experiment design to optimize enzyme treatment conditions for the degradation of commercial biodegradable mulch films

| Experiment | Film | Ridge size length × width (m) | Film spreading period (days) | Temperature at  enzyme treatment (℃) | Enzyme concentration  (U) | Enzyme amount  (mL/m^2^) | Replication | Date of  film spreading | Date of  enzyme treatment |
| --- | --- | --- | --- | --- | --- | --- | --- | --- | --- |
| 1 | A | 5 × 1 | 29 | 20 | 1, 3, 6 | 200 | 4 | September 8, 2014 | October 7, 2014 |
| 2 | A | 6 × 1 | 32 | 31 | 6 | 24, 60, 120* | 4 | June 19, 2015 | July 21, 2015 |
| 3 | B | 8 × 1 | 54 | 14 | 5.6 | 120 | 3 | September 1, 2016 | October 2, 2016 |
| 4 | B | 8 × 1 | 48 | 14 | 6 | 120 | 3 | September 2, 2017 | November 13, 2017 |
| 5 | C | 8 × 1 | 48 | 14 | 6 | 120 | 3 | September 2, 2017 | November 13, 2017 |
| 6 | A | 15 × 1 | 35 | 27 | 3, 6 | 100 | 3 | August 24, 2015 | September 28, 2015 |

*1) the entire surface of the ridge was sprayed with enzyme solution (PaE 6 U, with or without 2% SOFTON) at 120 mL/m^2^, the ridge was compartmentalized with 50-cm widths and the enzyme solution was applied at 1-compartment intervals (total 60 mL/m^2^) the ridge was alternately compartmentalized with widths of 50 cm and 10 cm, with only the 10-cm wide region treated with enzyme (total 24 mL/m^2^).

**Supplementary Table S3.** Molecular weight of neat polymers used in this study

| Material | Mn | Mw | Mw/Mn | Samples for analysis |
| --- | --- | --- | --- | --- |
|  | (×10^4^) | (×10^4^) |  |  |
| PBAT | 5.30 | 11.0 | 2.1 | Resin pellets |
|  | 1.63 | 10.5 | 6.4 | Heat-pressed film |
| PBSA | 1.13 | 25.8 | 22.7 | Heat-pressed film |
| PBS | 1.31 | 26.8 | 20.4 | Heat-pressed film |

**Supplementary Table S4.** Monodisperse polystyrene used as reference standard

| Peak molecular weight values (Mp) | Product number | Manufacturer |
| --- | --- | --- |
| 3,787,000 | Batch No. 20146-13 | Polymer Laboratories Ltd., Church Stretton, UK |
| 1,090,000 | F-128(TS-206) | TOSOH |
| 538,000 | Batch No. 20139-25 | Polymer Laboratories |
| 355,000 | F-40(TS-85) | TOSOH |
| 190,000 | F-20(TS-140) | TOSOH |
| 66,000 | 5040-35134 | GL Sciences Inc., Tokyo, Japan |
| 37,900 | F-4(TS-202) | TOSOH |
| 18,100 | F-2(TS-504) |  |
| 5,560 | A-5000(TS-518) |  |
| 2,630 | A-2500(TS-502) |  |
| 1,050 | A-1000(TS-501) |  |
| 500 | A-500(TS-505) |  |

■**Safety evaluation of enzyme solutions by external organizations**

A culture filtrate of the yeast *P. antarctica* (PaE, 6 U/ml) was used at the Biosafety Research Center, Inc. (Shizuoka, Japan) in 1) a bacterial reverse mutation test, 2) an acute oral toxicity test in rats, and 3) a 24-h occluded human-patch test, according to 1) the Guidelines for Designation of Food Additives and Revision of Standards for Use^9^, 2) the Guidelines for Testing of Chemicals 420^10^, and 3) Kawamura et al.^11^, respectively. The absence of mutagenicity, acute toxicity, and skin irritation has also been confirmed.

REFERENCES

1. Sato, S. et al. Degradation profiles of biodegradable plastic films by biodegradable plastic-degrading enzymes from the yeast *Pseudozyma antarctica* and the fungus *Paraphoma* sp B47-9. *Polym Degrad Stabil* **141**, 26–32 (2017). https://doi.org/[10.1016/j.polymdegradstab.2017.05.007](https://doi.org/10.1016/j.polymdegradstab.2017.05.007)

2. Kitamoto, H. K. et al. Phyllosphere yeasts rapidly break down biodegradable plastics. *AMB Express* **2011**, *1* (1), 44. https://doi.org/10.1186/2191-0855-1-44

3. Suzuki, K. et al. Purification, characterization, and cloning of the gene for a biodegradable plastic-degrading enzyme from *Paraphoma*-related fungal strain B47-9. *Appl Microbiol Biot* **2014**, *98* (10), 4457-4465. https://doi.org/10.1007/s00253-013-5454-0

4. Watanabe, T. et al. A UV-induced mutant of *Cryptococcus flavus* GB-1 with increased production of a biodegradable plastic-degrading enzyme. *Process Biochem* **2015**, *50* (11), 1718-1724. https://doi.org/10.1016/j.procbio.2015.07.005

5. Suzuki, K. et al. Affinity purification and characterization of a biodegradable plastic-degrading enzyme from a yeast isolated from the larval midgut of a stag beetle, *Aegus laevicollis*. *Appl Microbiol Biotechnol* **2013**, 97 (17), 7679-88. https://doi.org/10.1007/s00253-012-4595-x

6. Ueda, H. et al. Cutinase-like biodegradable plastic-degrading enzymes from phylloplane yeasts have cutinase activity. *Biosci Biotechnol Biochem* **2021**, *85* (8), 1890-1898. https://doi.org/10.1093/bbb/zbab113

7. Shinozaki, Y. et al. Biodegradable plastic-degrading enzyme from *Pseudozyma antarctica*: cloning, sequencing, and characterization. *Appl Microbiol Biot* **97**(7), 2951–2959 (2013). https://doi.org/[10.1007/s00253-012-4188-8](https://doi.org/10.1007/s00253-012-4188-8)

8. Shinozaki, Y. et al*.* Enzymatic degradation of polyester films by a cutinase-like enzyme from *Pseudozyma antarctica*: surface plasmon resonance and atomic force microscopy study. *Appl Microbiol Biot* **97**, 8591–8598 (2013). https://doi.org/[10.1007/s00253-012-4673-0](https://doi.org/10.1007/s00253-012-4673-0)

9. https://www.mhlw.go.jp/english/topics/foodsafety/foodadditives/dl/tenkabutu-shiteikijunkaiseishishin-english.pdf

10. https://ntp.niehs.nih.gov/iccvam/suppdocs/feddocs/oecd/oecd_gl420.pdf

11. Kawamura, T.et al. Basic studies on the standardization of the patch test (in Japanese), Jpn J Dermatol 80, 301-314 (1970). https://doi.org/10.14924/dermatol.80.301
